# 

**Published:** 1882-06

**Authors:** 


					﻿VOLIL
JONE, 1882.
NO. 6.-
JRHE
[1OMCEOPATHIC pHY^lClAp,
A MONTHLY JOURNAL OF MEDICAL SCIENCE.
“ JZagoia est' verilds, el prcevalebit."
ZE. CT. LEM M. ZD.
EDITOR.
CONTENTS:
(See inside of Cover0-
PHILADELPHIA:
No. 3109 CHESTNUT STREET. }
L O W D O 3ST.
ALFRED HEATH & CO;, Sole Agents for Great Britain,
114 Ebury Street, Eatop Square.
Yearly Subscription, $2.00, invariably in advance.
Entered at the Philadelphia Post-Office, as Second Class Mail Matter.
				

